# Per-cutaneous dilatation tracheostomy (PCTD) in COVID-19 patients and peri-tracheostomy care: A case series and guidelines

**DOI:** 10.12669/pjms.36.7.3518

**Published:** 2020

**Authors:** Ehtesham Khan, Shankar Lal, Junaid Hashmi, Jubil Thomas, Muhammad Anwar Malik

**Affiliations:** 1 Dr. Ehtesham Khan, CAI MSc, FCPS, FCARCSI, FJFI CMI, EDIC, DMMD, Dip Pain RCSI. Department of Anaesthesia and Critical Care Medicine, Our Lady of Lourdes Hospital, Drogheda, Ireland; 2 Dr. Shankar Lal, FCPS. Department of Anaesthesia and Critical Care Medicine, Our Lady of Lourdes Hospital, Drogheda, Ireland; 3 Dr. Junaid Hashmi, FCIA. Department of Anaesthesia and Critical Care Medicine, Our Lady of Lourdes Hospital, Drogheda, Ireland; 4 Dr. Jubil Thomas, MBBS, MD, FCARCSI, FJFICMI, CCST, CHCM, Department of Anaesthesia and Critical Care Medicine, Our Lady of Lourdes Hospital, Drogheda, Ireland; 5 Dr. Muhammad Anwar Malik, MBBS, FCPS, FCAI, Dip Pain RCSI Department of Anaesthesia and Critical Care Medicine, Our Lady of Lourdes Hospital, Drogheda, Ireland

**Keywords:** Percutaneous dilatational tracheostomy, COVID-19 patients, aerosol generating

## Abstract

**Background & Objective:**

COVID 19 patients with severe respiratory failure may require prolonged mechanical ventilation. Placement of a tracheostomy tube often becomes necessary for such patients. The steps of tracheostomy procedure and post tracheostomy care of these patients can be classified as aerosol generating. We wish to highlight our modified technique to address these issues.

**Methodology:**

We performed percutaneous dilation tracheostomy in three clinically challenging COVID-19 patients in our ICU and developed guidelines aiming to minimise aerosolisation during and after the tracheostomy procedure to safeguard healthcare workers.

**Results:**

Percutaneous tracheostomy was performed by a team of three experienced anaesthetists and an ICU nurse.

**Conclusion:**

The decision of surgical or percutaneous tracheostomy should be dependent on the experience of the tracheostomy performer, health-care worker safety, resource availability, and patient-centred care. We believe our modified strategic approach of brief bronchoscopy, minimum PEEP and gas flows and step-wise planned approach for PCDT offers an extra level of safety to healthcare workers.

## INTRODUCTION

COVID-19 patients with severe respiratory failure may require prolonged mechanical ventilation. Placement of a tracheostomy tube often becomes necessary for such patients.The actual tracheostomy procedure as well as post tracheostomy care of these patients involves multiple steps which can be classified as Aerosol Generating. Also, there is no consensus regarding which approach, Surgical or PCDT, is more suitable or whether to perform ‘early’ or ‘delayed’ tracheostomy considering the infectious risk. Initial data suggested that patients clear the SARS-CoV-2 virus on average within 2-3 weeks after onset of symptoms/diagnosis but a few recent articles report patients testing positive for over 70 days.^1^ Also early tracheostomy insertion has not been proven to be superior to a delayed **one.^2^** We performed three percutaneous tracheostomies in our intensive care unit and developed guidelines addressing the technique to minimize aerosolization during procedure and critical steps during post-tracheostomy care and transfer in ICU and ward.

## CASES REPORT

### Cases description

All tracheostomies were percutaneous because PCDT carries lesser risk of aerosolization, bleeding and require no or minimum use of diathermy as compared to surgical tracheostomy. We performed all PCDT when our patients showed evidence of minimum requirement of PEEP and FiO_2_ along with clinical evidence of ability to tolerate interruption of ventilation.^2^

### Case-1

A 54-year-old morbidly obese male, known hypertensive and diabetic, presented with a short history of severe respiratory distress and persistent hypoxemia not responding to oxygen therapy and required intubation and ventilation. Multiple attempts to wean the patient off the ventilator failed. After a multi-disciplinary discussion PCDT was performed after 31 days of intubation.

### Case-2

A 60-year-old morbidly obese male and known case of HTN & DM presented with severe hypoxemia, hypotension and required urgent intubation and mechanical ventilation. Following multiple failed weaning trials from the mechanical ventilator; the decision was made to proceed for tracheostomy at Day 27.

### Case-3

A 64-year-old female, with the background history of HTN, DM, severe cervical ankylosing spondylitis and limited neck mobility, presented with severe hypoxemia and ARDS. The patient required intubation and ventilation. After multiple failed attempts to wean, the decision to proceed with PCDT was also made at Day 27.

### Procedure

Percutaneous tracheostomies were performed in the negative pressure rooms of intensive care with the number of people restricted to four; three experienced anaesthetists (one bronchoscopist, one operator and one assistant) and an ICU nurse. Every member of the team had proper personal protective equipment. To decrease the airway secretions and reduce the risk of aerosolization, we established modified technique as described below,

- Each patient received two aliquots of 200mcg glycopyrrolate intravenously, 60 minutes and 30 minutes prior to the procedure. Patients received 100% Fio2 ten minutes prior to the start of the procedure which was continued till the end of the procedure.
- We ensured full muscle relaxation and adequate sedation to avoid any cough and gag response while performance of the procedure.
- To shorten the duration of procedure, the patients’ position was optimised by neck extension and by the placement of a rolled towel under the shoulders.
- A neck ultrasound was performed to visualise the positions of the trachea, tracheal rings and blood vessels. 5 ml of Local anaesthetic (2 % lignocaine with 1/200000 adrenaline) was infiltrated at the site of incision.
- Ventilator was then put on standby and a brief bronchoscopy was performed by the experience anaesthetist using a swivel connector attached to an endotracheal tube.
- The endotracheal tube was pulled back till the cuff was visualized at the glottis ventilation was then resumed.
- PCDT was performed with guidewire dilator technique starting with puncture of the anterior tracheal wall, seldinger technique, dilatation and cannula positioning.
- Ventilation was paused during the insertion of the dilator and insertion of the tracheal tube.
- The complete process of insertion and correct position of tracheostomy tube was monitored by bronchoscopy.
- The assistant covered the tracheostomy site during dilation of tracheostomy stoma insertion site to minimise aerosolization. The position of the tracheostomy tube was confirmed with bronchoscope, chest rise and capnography.
- The endotracheal tube was subsequently removed with clamp still on.

We ensured minimum gas flows and PEEP following performance of per cutaneous tracheostomy. Case one required placement of a PORTEX® UniPerc® Adjustable Flange Extended-Length tracheostomy Tubes as the Portex percutaneous tracheostomy tube didn’t have enough length to reach the trachea, as patient was morbidly obese. The other two patients were successfully managed with Portex® ULTRAperc® tracheostomy tube.

## DISCUSSION

Timely tracheostomy may allow for patients to be weaned off sedation faster and moved to intermediate care wards, freeing up ICU resources but False-negative PCR test results are an additional concern and therefore reasonable measures to protect staff and the patients should be continuously practiced.^1^,^3^ The tracheotomy and post tracheostomy care of such patients should be meticulously planned and performed in a negative pressure room facility (wherever available), to address these issues, we established guidelines and proforma for peri-tracheostomy care of such patients involving anaesthesiologists, ENT surgeons, tracheostomy nurses, Speech and Language Therapists & Physiotherapists. Each patient had a careful clinical review before being considered for tracheostomy.

On the day of the procedure, we ensured complete barrier precautions for the safety of all associated healthcare providers involved in the procedure. Standard PPE were donned by each person under a ‘buddy check’ programme. All necessary equipment was pre-arranged into sterile packs in an anteroom of negative pressure ICU suite. We reviewed the availability of all necessary equipment and scrutinised the steps as per the checklist and our organisational guidelines beforehand.

Each of these three percutaneous tracheostomies took around 25 minutes on average. Compared with a surgical tracheostomy, PCDT potentially reduces the risk of surgical site infection and rarely requires transfer to the theatre.^2^,^4^ We avoided the modified technique wherein the bronchoscope is passed by the side of endotracheal tube after changing the ETT to a smaller size.^5^-^7^ In our opinion this technique carries an increased risk of aerosolization as it requires changing ETT to smaller size and then passing bronchoscope by side of ETT which may not provide an optimum seal especially on high PEEP.

### Guidelines

The tracheostomy after-care of COVID-19 patients differs from routine care tracheostomy because of a high risk of transmission of infection due to Aerosolisation. Routine tracheostomy specific Aerosol Generating Procedures (AGPs) include tracheal open suctioning, tracheostomy changes and sputum induction^5^-^7^, but other interventions like chest physiotherapy, inner cannula changes and nebulisation may also increase the likelihood of coughing and sputum production.^5^ There are seven crucial steps involved^6^, and the frequency of each of these interventions should be reviewed and re-evaluated as needed to reduce clinical risk to the patient as well as to protect staff (Table-I).

**Table-I:** Describing the stepwise approach of Post-tracheostomy care in ICU and Ward with emphasis on minimisation of aerosolization.

Steps	Recommendations
Stoma dressing/Cleaning	Assess daily as regular cleaning reduces risk of infection. Can be performed with the ties in situ to reduce the risk of accidental de-cannulation and the need for two staff members
Tracheostomy tapes/ties in ICU	IN ICU: Change daily and additionally as needed.
	IN WARD: Change as required
Inner cannula changes	Check every 6 hours regardless of tracheostomy type and change every 12 hours. If patient has thick secretions more regular checking of the inner cannula is required.
	Ensure two staff are present in the changing of the inner cannula. If patient is on the ventilator, it should be put on STAND-BY during change. Disposable inner cannulas should be replaced as per the manufacturer’s guidelines. They should not be cleaned and reused.
Cuff pressure Monitoring	Ensure the cuff remains fully inflated (15-30 cm H20^)8^, to reduce the risk of virus aerosol. This should be re-checked post position changes and after suctioning.
One-Way Speaking Valve and Tracheostomy Cap Trial	As indicated
First Tube Change	7-10 days9, (also review case by case basis)
Subsequent Tube Change	30-day interval^9^, (review case by case basis)

Positive pressure ventilation also increases the potential for aerosol risks to staff,^7^ and staff taking care of patients receiving positive pressure ventilation should don appropriate PPE. A cuff inflated, closed system is most likely to prevent cross-contamination of staff, equipment and other patients and therefore closed in-line suction is recommended.^8^

In the wards a regular Multi-disciplinary tracheostomy ward round should be done. A daily record of all tracheostomy related care/intervention/events should be maintained. All tracheostomy care interventions should be treated as AGPs. A simple face mask should be applied over the face of the patient once the cuff is deflated to minimize droplet spread.

Any tracheostomy tube change should be discussed by the clinical team to outline the potential risks versus the benefits of this AGP. The procedure should be performed with full PPEs, and preferably in a single room with negative pressure facility. Ensure availability of all emergency equipment and drugs before the start of a procedure. All patients should be trialled on dry oxygen via HME filter as first line intervention.^5^ For routine tracheal suctioning a closed, inline suction with HME filter should be preferred to reduce the risk of aerosolization.^9^

Initially we proposed a simple system, for spontaneously breathing patients with tracheostomy in–situ, which had a Closed Suction Unit, HME Filter and Swedish nose for oxygen supply (Fig-I). Although simple, this circuit is ‘heavy’ and can cause drag on the tracheostomy tube.

**Fig.1:**
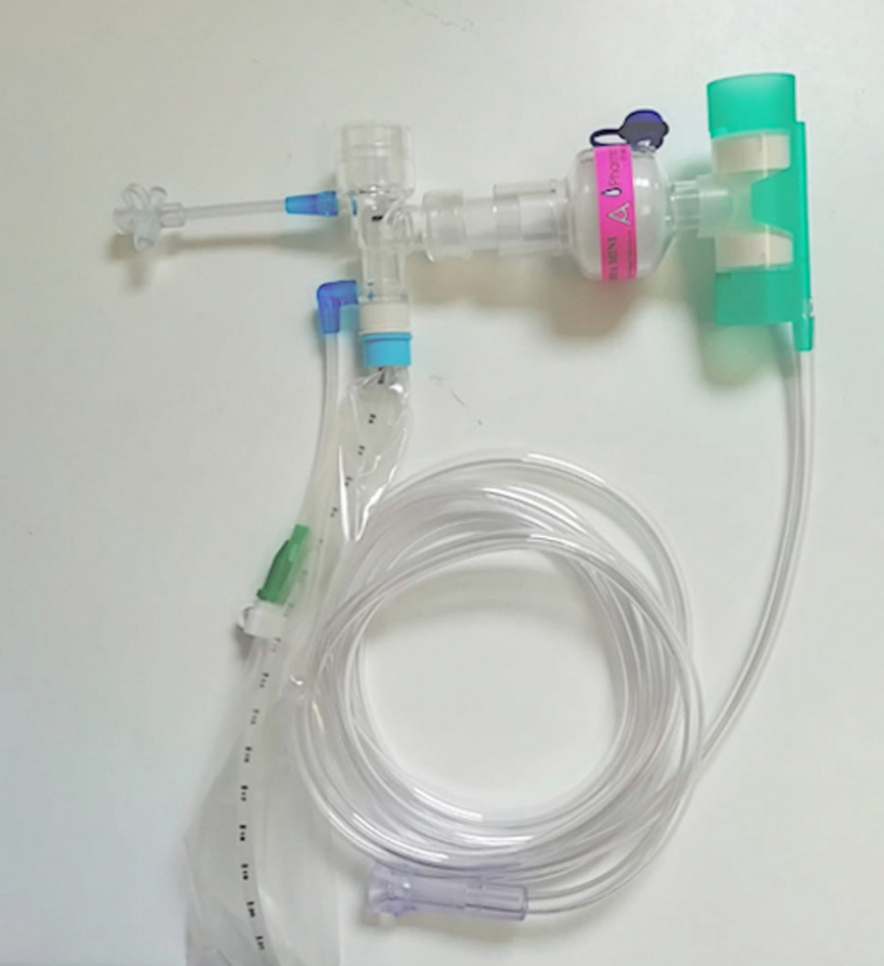
Alternate to Kelly circuit for spontaneously breathing patients. It comprises of Swedish Nose, HME filter and closed suction unit.

We eventually used a novel circuit called Kelley Circuit (Fig.II). The Kelley Circuit combines the ProTrach® XtraCare™ HME with an electrostatic filter with a closed-circuit suction system.^10^ In our experience this circuit is more compact and light-weight and therefore will cause less drag.

**Fig.2:**
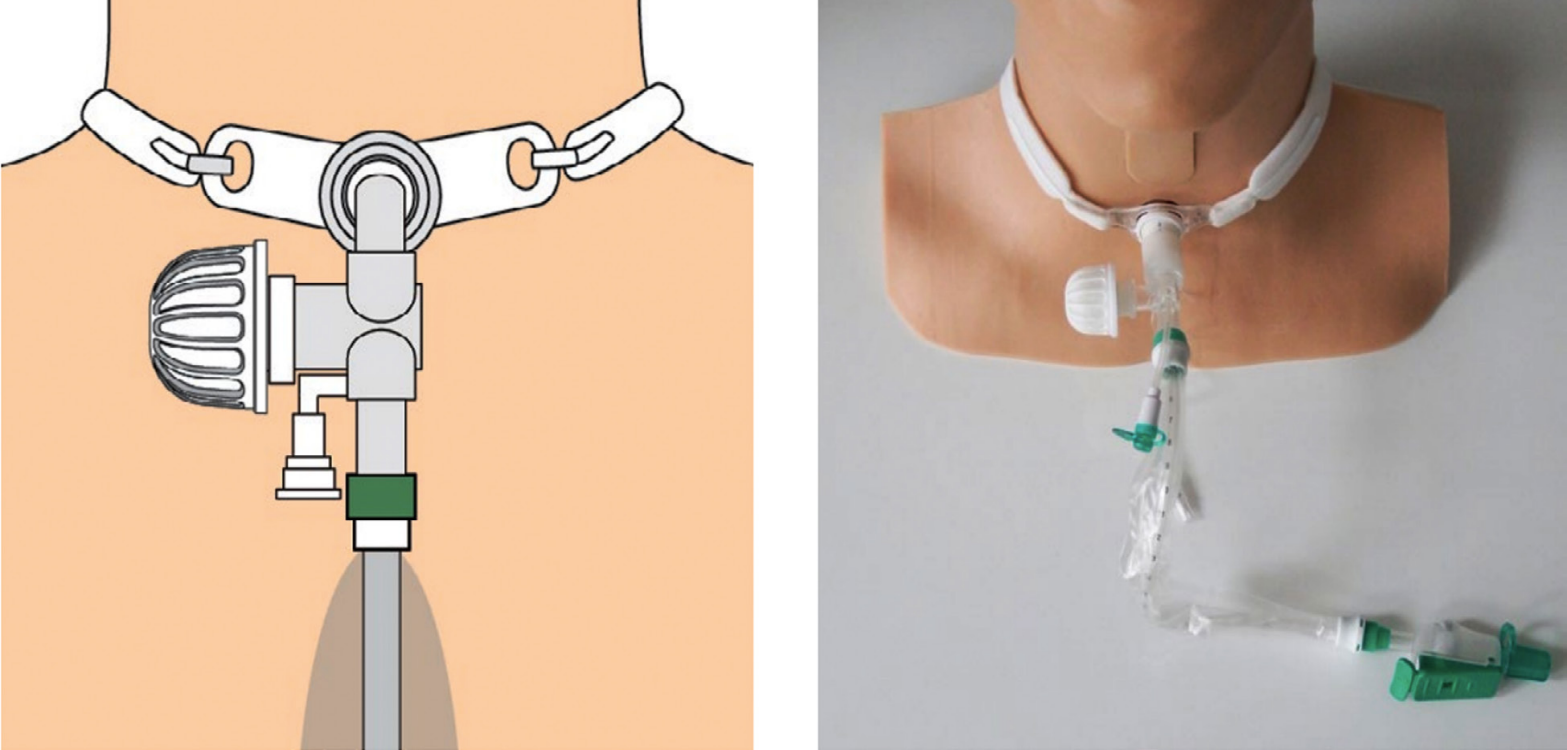
The Kelley Circuit with a closed circuit suction system attached to the ISO 15 hub of the tracheostomy tube and the ProTrach XtraCare attached to the ventilator hub on the side.

A surge of COVID-19 patients can overwhelm hospitals with a possibility of many requiring mechanical ventilation and possible tracheostomy. The decision of surgical or percutaneous tracheostomy should be dependent on the experience of the tracheostomy performer, health-care worker safety, resource availability, and patient-centered care. Proper and acceptable guidance for performance and post tracheostomy care is crucial and should be established in advance. We believe our modified strategic approach for PCDT offers an extra level of safety to healthcare workers.

### Authors’ Contribution:

**EK:** Performance of PCDT, Literature search, and Initial Write-up and Guidelines.

**SL:** Data collection, Writing as co-author, Guidelines.

**JH:** Writing as co-author, Literature Search, Guidelines.

**JT:** Performance of PCDT, Literature search.

**MA:** Literature search, Guidelines, and Final Write-up.
